# Factors influencing hospital length of stay in febrile neutropenia: A retrospective cohort study of Turkish patients with solid tumors

**DOI:** 10.1097/MD.0000000000043105

**Published:** 2025-07-04

**Authors:** Ruhper Cekin, Didar Senocak, Sener Cihan

**Affiliations:** a Department of Oncology, Okan University Hospital, Istanbul, Türkiye; b Department of Oncology, University of Health Sciences, Sultan Abdulhamid Khan Training and Research Hospital, Istanbul, Türkiye; c Istinye University Hospital, Medikalpark Gaziosmanpasa, Department of Oncology, Istanbul, Türkiye.

**Keywords:** febrile neutropenia, hospitalization, length of stay, malignity, mortality, solid tumor

## Abstract

Febrile neutropenia (FN) is a severe complication of chemotherapy, associated with substantial mortality and financial burden. The purpose of this study was to assess the association between hospital length of stay (LOS), supportive therapies, and antibiotic regimens in patients with FN, with a specific focus on the Turkish population. Eighty adult patients with solid tumors were enrolled. Patients received empirical antibiotic therapy within 2 hours of presentation. Data were collected on clinical and demographic variables, including LOS, fever duration, Multinational Association of Supportive Care in Cancer Risk Index scores, and laboratory parameters. The mean hospital LOS was 6.09 ± 3.62 days. Sulperazon use was significantly associated with a shorter LOS compared to meronem and tazocin (*P* < .001). Patients receiving filgrastim had a longer LOS compared to those who did not (*P* = .042). Correlation analysis revealed strong positive associations between LOS and febrile days (*R* = 0.624, *P* < .001), febrile days during hospitalization (*R* = 0.711, *P* < .001), and filgrastim administration days (*R* = 0.722, *P* < .001). Multivariate analysis confirmed that sulperazon use reduced LOS by 1.271 days (*P* = .048), while prolonged filgrastim use was linked to longer stays (*P* < .001). These findings highlight the critical role of antibiotic selection and supportive care in managing hospitalization duration for patients with FN. The combination of certain treatments and antibiotics plays a significant role in determining the duration of hospital stays, highlighting factors to consider in patient management and treatment planning.

## 1. Introduction

Febrile neutropenia (FN), characterized by fever and an absolute neutrophil count (NC) below 500 cells/mm³, represents a critical complication in oncology patients undergoing chemotherapy.^[1,2]^ This condition, caused by a diminished immune response to infections, demands immediate medical attention to reduce associated morbidity and mortality. Globally, FN remains a pivotal concern, especially in oncology and hematology settings, where it is a frequent reason for hospitalization.^[3]^ Early initiation of appropriate empiric antibiotic therapy is widely recognized as a cornerstone of FN management, significantly reducing mortality and improving patient outcomes.^[4]^

While these international perspectives offer valuable guidance, it is equally important to consider region-specific data when evaluating treatment outcomes in FN. In Türkiye, adult studies investigating FN are limited, particularly among patients with solid tumors. A notable study by Gunalp et al assessed adult oncology patients with FN admitted to the emergency department and identified independent predictors of poor outcomes, including thrombocytopenia, pulmonary infiltration, hypoproteinemia, tachypnea, and a Multinational Association of Supportive Care in Cancer (MASCC) score <21.^[5]^ These findings offer valuable insights into risk stratification and early clinical intervention. Similarly, other adult-focused studies have explored FN-related complications in hematological malignancies and highlighted the burden of secondary infections, yet there remains a scarcity of research specifically targeting the solid tumor population in Türkiye.^[6,7]^ In contrast, the Turkish pediatric literature has more extensively addressed FN characteristics. For instance, a study on children with leukemia treated under the BFM protocol revealed that FN episodes were most prevalent during the consolidation phase, unlike international findings where the induction phase predominates.^[8,9]^ This discrepancy underscores the influence of chemotherapy regimens and local microbial patterns on FN occurrence and outcomes. Moreover, elevated C-reactive protein (CRP) levels have been consistently associated with bacterial infections in pediatric populations, supporting their role in infection risk stratification.^[6]^ These data collectively emphasize the need for region-specific investigations to optimize antibiotic strategies and improve patient outcomes in both adult and pediatric oncology settings.

The hospital length of stay (LOS) is an essential marker of clinical severity and resource utilization in FN management.^[10]^ It is reported that prolonged LOS increases the risk of healthcare-associated infections and delays subsequent cancer treatments, negatively affecting both patient prognosis and healthcare system efficiency.^[11]^ Furthermore, multidrug-resistant bacterial infections, high-dose chemotherapy regimens, and extended durations of neutropenia are consistently identified as predictors of prolonged LOS. In addition, interventions targeting LOS reductions, such as optimizing antibiotic use and supportive care strategies, can mitigate the economic burden of FN, improve bed turnover, and enhance treatment outcomes.^[10]^ While there is substantial evidence on the impact of timely antibiotic administration on mortality and intensive care unit admission rates in patients with FN, its effect on LOS remains inconclusive. Some studies suggest that early empiric antibiotic therapy significantly reduces LOS, while others show no consistent association.^[10,12]^

This study aims to bridge the knowledge gap by evaluating the interplay between antibiotic choice and hospital LOS in FN patients, focusing on factors influencing clinical severity and resource utilization in a tertiary care setting. By analyzing regional microbial trends and clinical outcomes, we seek to provide actionable insights for improving FN management strategies.

## 2. Materials and methods

This retrospective cohort study was approved by the Ethics Committee of the University of Health Sciences, Istanbul Prof Dr Cemi̇l Tascioglu City Hospital, Istanbul, Türkiye, (Study Protocol Number: 29/12/2020-486707771 514.10), and was performed in accordance with the Declaration of Helsinki. Written informed consent was obtained from all participants. Patients with solid tumors who were admitted to the oncology clinic for 26 months, from September 2021 to November 2023, were enrolled in the study. The demographic and clinical features and blood test results of participants were recorded.

### 2.1. Participants

Eighty adult patients with solid tumors, meeting the International Classification of Diseases-10 and Infectious Diseases Society of America criteria for FN^[13]^ NC < 500 cells/mm³ and a fever of 38.3°C occurring once within 24 hours or 38°C sustained for 1 hour, were enrolled in the study. Antibiotic therapy was initiated within 2 hours of their presentation to the emergency department.

Patients’ data were recorded in a database that included information about clinical and demographic characteristics of the patients including LOS, age, body mass index (BMI), fever, chemotherapy cycle, febrile days, febrile days during hospital stay, filgrastim days, and blood test parameters such as WBC, NC, hemoglobin, lymphocyte count, platelet count (PLT), and CRP.

Additionally, the MASCC Risk Index, which is a scoring system used to predict the risk of serious complications during febrile neutropenic episodes, was utilized.^[14]^ It includes clinical factors such as the severity of symptoms, type of cancer, presence of hypotension, dehydration, and other comorbidities. A higher score indicates a lower risk of complications, helping physicians determine whether outpatient treatment may be appropriate. Individuals with a MASCC score >21 were also excluded.

None of the participants had any anatomic or genetic abnormalities, endocrine disorders, or chronic inflammatory diseases. Patients <18 years of age, those diagnosed with hematologic malignancies or chronic organ failure, patients who received antibiotics >2 hours after presentation, those with a defined source of infection, and those with positive blood culture results were excluded from the study.

### 2.2. Treatment protocol and management

Empirical antibiotic selection was determined based on the physician’s clinical judgment and preference. Only the first episode of FN was included for patients with recurrent episodes, ensuring consistency in data collection. The primary outcome of the study was the LOS after the onset of FN. Chemotherapy protocols were administered in alignment with established international guidelines. For supportive care, patients weighing ≤60 kg were prescribed 30 million units of Filgrastim, while those weighing >60 kg received 48 million units of Filgrastim.

### 2.3. Statistical analysis

R, an open-source software environment for statistical computing and graphics developed by the R Foundation for Statistical Computing (version 4.4.2, 2024; Vienna, Austria), was employed for all statistical computations and model fitting. Descriptive statistics were used to summarize categorical variables as frequencies and percentages, and numerical variables as means, SDs, minimums, maximums, and medians. The normality of numerical variables was assessed using the Shapiro–Wilk test and plots such as histograms and Q-Q plots were used for visual inspection. For univariate analysis, the Mann–Whitney *U* test was used to compare 2 groups, and the Kruskal–Wallis test was used to compare 3 or more groups, with post hoc pairwise comparisons performed using the Dunn test. For correlation analysis of numerical variables, Spearman rank correlation was applied to evaluate monotonic relationships. For multivariate analysis, a Generalized Linear Model with a Poisson distribution and a log link function was applied to model the “Length of Stay” variable. The statistical alpha significance value was accepted as *P* < .05.

## 3. Results

The demographic and clinical characteristics of the study population were shown in Table 1.

**Table 1:** The demographic and clinical characteristics of the study population.

Variables	Mean ± SD	Median (Q25–75)	Min	Max
Length of stay	6.09 ± 3.62	5.00 (4.00–7.00)	1.00	26.0
Age	61.5 ± 14.3	64.0 (56.8–70.0)	18.0	86.0
BMI	26.0 ± 4.57	25.6 (23.0–28.3)	18.0	41.0
Admission CRP	189 ± 114	166 (99.2–263)	12.4	535
Admission WBC	772 ± 451	670 (428–992)	120	2000
Initial fever	38.5 ± 0.295	38.5 (38.3–38.6)	38.0	39.5
Chemotherapy cycle	2.85 ± 1.94	2.00 (1.00–4.00)	1.00	10.0
Filgrastim days	3.56 ± 2.56	3.00 (2.00–4.00)	0	18.0
MASCC score	17.6 ± 2.20	18.0 (15.0–20.0)	13.0	22.0
Total number of chemotherapy	1.29 ± 0.508	1.00 (1.00–2.00)	1.00	3.00
Total febrile days	3.11 ± 1.96	3.00 (2.00–4.00)	0	12.0
Febrile days during hospital stay	1.93 ± 1.63	2.00 (1.00–2.00)	0	10.0
HGB	9.10 ± 2.39	8.80 (7.70–10.8)	0.900	14.0
PDW	15.8 ± 2.05	16.1 (15.8–16.4)	0	17.1
PLT	93492 ± 99562	67,000 (36,000–112,250)	131	517,000
Lymphocyte	440 ± 398	300 (158–600)	0	2060
Discharge WBC	6394 ± 4767	5030 (2945–8842)	110	20,350
Discharge neutrophil count	4739 ± 3821	3730 (1635–7272)	0	15,870

BMI = body mass index, CRP = C-reactive protein, HGB = haemoglobin, MASCC = Multinational Association of Supportive Care in Cancer, PDW = platelet distribution width, PLT = platelet count, WBC = white blood cell count.

The mean age of the participants was 61.5 ± 14.3 years. Regarding gender distribution, 56% of the participants were male and 44% female. The BMI had a mean of 26.0 ± 4.57, ranging from 18.0 to 41.0. The mean length of hospital stay was 6.09 ± 3.62 days and ranged between 1.00 and 26.0 days. Initial fever measurements averaged 38.5 ± 0.29°C and admission CRP levels had a mean of 189 ± 114 mg/L. Admission WBC counts averaged 772 ± 451 × 10³/μL. The mean number of febrile days was 3.11 ± 1.96. Febrile days during the hospital stay had a mean of 1.93 ± 1.63 days. Filgrastim administration lasted an average of 3.56 ± 2.56 days, ranging from 0 to 18 days. Hematological findings included a mean hemoglobin of 9.10 ± 2.39 g/dL. Lymphocyte counts averaged 440 ± 398 × 10³/μL, ranging from 0 to 2060 × 10³/μL. PLT counts had a mean of 93,492/mm³. The MASCC score averaged 17.6 ± 2.20, and ranged between 13.0 and 22.0.

Among the antibiotics used, tazocin was the most frequently administered (48%), followed by sulperazon (31%) and meronem (21%). Most patients (94%) received at least 1 dose of filgrastim during their treatment, while 62% did not receive filgrastim prophylaxis. The majority of patients (80%) did not receive radiotherapy in the past 15 days, while 20% did.

The most common diagnosis among the patients was small cell lung cancer (SCLC), observed in 25 patients (31.25%), followed by gastric cancer, diagnosed in 14 patients (17.5%). Gastrointestinal cancers-including gastric, rectal, pancreatic, and colorectal sarcomas-accounted for a significant portion of the cohort (43.75%). Gynecologic malignancies such as ovarian and endometrial cancers were observed in 6.25% of patients. Other diagnoses included breast cancer (8.75%), non-SCLC (7.5%), and leiomyosarcoma (1.25%).

Regarding chemotherapy protocols, the most frequently administered regimen was cisplatin-etoposide (27.5%), primarily used in patients with SCLC. Gastrointestinal cancer regimens, including FLOT, FOLFIRI, and FOLFIRINOX, were also commonly prescribed, accounting for approximately 30% of treatments. Fluoropyrimidine-based combinations with targeted agents (e.g., FOLFIRI-cetuximab, FOLFIRI-aflibercept, FOLFIRI-bevacizumab) were used in a smaller subset. Platinum-taxane combinations (e.g., carboplatin-paclitaxel, cisplatin-pemetrexed) and anthracycline-based regimens (e.g., AC regimen [doxorubicin and cyclophosphamide]) were less frequently observed. Other regimens, including monotherapy with docetaxel or irinotecan, were administered to a limited number of patients.

**Figure 1.**
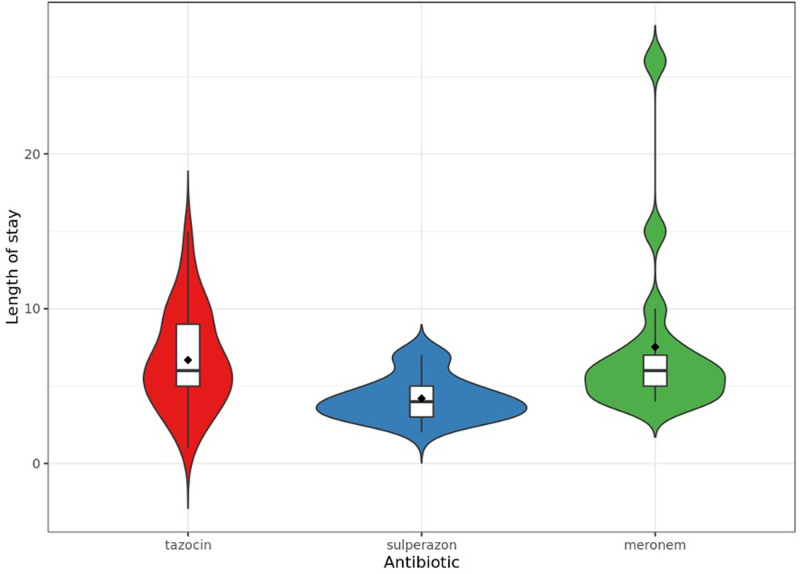
The distribution of length of stay depending on antibiotics.

Univariable analysis by LOS was performed in the study to evaluate the clinical characteristics of the patients (Table 2). The median length of hospital stay varied significantly among patients receiving different antibiotics (*P* < .001). Patients receiving either meronem or tazocin had a median LOS of 6.00 days, while those treated with sulperazon had a shorter median LOS of 4.00 days. post hoc analysis showed that sulperazon was associated with significantly shorter LOS compared to both meronem (*P* = .0010) and tazocin (*P* = .0001), whereas the difference between meronem and tazocin was not statistically significant (*P* = .4475). Figure 1 depicts the distribution of LOS depending on antibiotics. Patients who received filgrastim had a significantly longer median LOS (5.00 d) compared to those who did not (3.00 d; *P* = .042). No significant difference was observed in the LOS between those who received filgrastim prophylaxis and those who did not (*P* = .21). Patients with adverse FN) outcomes, described as death, had a longer median hospital stay (8.00 d) compared to those with favorable outcomes (5.00 d; *P* = .22). The LOS was not significantly associated with gender (*P* = .40) or recent radiotherapy within 15 days (*P* = .1). However, male patients had a slightly longer median stay (5.50 d) compared to female patients (5.00 d). Patients who had received radiotherapy had a median LOS of 4.50 days compared to 5.50 days for those who did not.

**Table 2:** Univariable analysis of clinical characteristics associated with length of hospital stay.

Variables		Mean ± SD	Median (Q25–75)	Min	Max	n	*P* value	Statistical test
Type of antibiotics	Tazocin	(6.68 ± 3.16)	6.00 (5.00–9.00)	1.00	15.0	38	<.001	Kruskal–Wallis
	Sulperazon	(4.20 ± 1.38)	4.00 (3.00–5.00)	2.00	7.00	25		
	Meronem	(7.53 ± 5.49)	6.00 (5.00–7.00)	4.00	26.0	17		
Filgrastim	No	(3.80 ± 1.30)	3.00 (3.00–4.00)	3.00	6.00	5	.042	Mann–Whitney
	Yes	(6.24 ± 3.68)	5.00 (4.00–7.00)	1.00	26.0	75		
Filgrastim profilaxisi	No	(6.02 ± 2.67)	6.00 (4.00–7.00)	1.00	15.0	50	.21	Mann–Whitney
	Yes	(6.20 ± 4.87)	4.50 (4.00–6.00)	2.00	26.0	30		
Mortality	Alive	(5.75 ± 2.68)	5.00 (4.00–7.00)	1.00	15.0	75	.22	Mann–Whitney
	Death	(11.2 ± 9.58)	8.00 (5.00–15.0)	2.00	26.0	5		
Gender	Male	(6.59 ± 4.28)	5.50 (4.00–7.00)	1.00	26.0	44	.40	Mann–Whitney
	Female	(5.50 ± 2.61)	5.00 (3.25–7.00)	2.00	13.0	36		
Radiotherapy	No	(6.41 ± 3.84)	5.50 (4.00–7.00)	2.00	26.0	64	.1	Mann–Whitney
	Yes	(4.81 ± 2.26)	4.50 (3.00–5.50)	1.00	10.0	16		

The correlation analysis revealed significant positive correlations between the length of hospital stay and febrile days (*R* = 0.624), *P* < .001), febrile days during hospital stay (*R* = 0.711, *P* < .001), and filgrastim administration days (*R* = 0.722, *P* < .001) (Table 3). Additionally, a moderate negative correlation was observed between the length of hospital stay and the MASCC score (*r* = −0.342), *P* < .01). PLT showed a weak negative correlation (*r* = −0.212, *P* = .06), which was borderline significant. Other variables, including admission CRP, admission WBC, age, BMI, discharge WBC, lymphocyte count, and initial fever, showed no significant correlation with the length of hospital stay (*P* > .05).

**Table 3:** The correlation analysis between the length of hospital stay and clinical characteristics.

Variable name	Median (min–max)	Correlation coefficient	*P* value
Filgrastim_days	Median: 3.00 (1.00–18.00)	0.554	<.001
Total number of febrile days	Median: 2.00 (0.00–12.00)	0.437	<.001
Number of febrile days during hospital stay	Median: 2.50 (1.00–7.00)	0.319	.003
Age (yr)	Median: 61.00 (18.00–86.00)	0.094	.40
BMI	Median: 25.60 (18.00–41.00)	−0.036	.75
Number of chemotherapy cycle	Median: 2.00 (1.00–10.00)	−.080	.48
Total_number_of_chemotherapy	Median: 1.00 (1.00–3.00)	0.198	.08
Admission CRP level	Median: 166.00 (12.40–535.00)	−.004	.97
HGB	Median: 8.80 (0.90–14.00)	−.017	.88
Lymphocyte count	Median: 300.00 (0.00–2060.00)	−.057	.61
Discharge WBC count	Median: 5030.00 (110.00–20,350.00)	0.009	.93
Discharge neutrophil count	Median: 3730.00 (0.00–15,870.00)	0.037	.74
PLT	Median: 67,000.00 (131.00–517,000.00)	−.211	.05
MASCC score	Median: 18.00 (13.00–22.00)	−.341	.00
Radiotherapy	Median: 0.00 (0.00–1.00)	−.183	.10

BMI = body mass index, CRP = C-reactive protein, HGB = hemoglobin, MASCC = Multinational Association of Supportive Care in Cancer, PLT = platelet count.

For the multivariate analysis, we selected variables based on the univariate analysis results and prior domain knowledge. The univariate analysis revealed significant correlations between LOS, the dependent variable, and several continuous variables, including filgrastim days, febrile days, and others. We also identified 1 significant categorical variable, sulperazon, which significantly impacted the LOS in a post hoc Kruskal–Wallis test. To build the multivariate model, we included the continuous variables filgrastim days, total number of febrile days, febrile days during hospital stay, MASCC score, and PLT, which showed meaningful correlations with the dependent variable. We excluded variables with weak or no significant relationship with the target, such as MASCC score, in an effort to create a more robust model. The antibiotic sulperazon variable, a categorical variable, was converted into a dummy variable (1 for sulperazon, 0 for other antibiotics) and added to the model to capture its effect on LOS. By focusing on these variables, we aim to understand the combined effects of treatments, symptoms, and antibiotics on the LOS, while controlling for the effects of other factors that may influence the outcome. A Generalized Linear Model with a Gaussian distribution and an identity link function for the analysis was utilized. This method was suitable for continuous dependent variables like LOS, which allowed us to assess the relationships between the dependent variable and several independent predictors simultaneously. The model showed that filgrastim days and sulperazon were significantly associated with LOS. Specifically, for sulperazon, the model estimated that using sulperazon reduces the LOS by approximately 1.271 days (*P* = .048), while filgrastim days have a positive relationship, meaning longer filgrastim treatments correlate with a longer stay (*P* = .000). Table 4 summarizes the results of the multivariate analysis.

**Table 4:** Multivariate analysis of clinical characteristics associated with length of hospital stay.

Variable	Coefficient estimate	Standard error	*z* value	*P* value
Filgrastim_days	0.066	0.022	2.757	.005
Total number of febrile days	0.061	0.064	0.979	.327
Febrile days during hospital stay	−.004	0.051	−.083	.934
Filgrastim	0.176	0.243	0.726	.468
MASCC_score	−.008	0.025	−.318	.750
PLT	−.000	−.000	−.903	.367
Sulperazon use	−.324	0.123	−.639	.008

MASCC = Multinational Association of Supportive Care in Cancer, PLT = platelet count.

## 4. Discussion

Our findings identified several significant factors influencing LOS, including the choice of antibiotics, the duration of filgrastim administration, and the number of febrile days. Notably, patients treated with sulperazon had a shorter LOS compared to those receiving meronem or tazocin. However, as this is an observational study, this association may reflect underlying differences in patient characteristics or prescribing preferences rather than a direct therapeutic advantage. In contrast, prolonged filgrastim administration was associated with extended LOS, highlighting the need for careful consideration when using supportive therapies in FN management. Additionally, we observed a strong positive correlation between LOS and febrile days, underscoring the critical role of managing persistent fever in optimizing patient outcomes and reducing hospitalization duration. These findings align with existing literature, further supporting the importance of individualized approaches to FN treatment. Our study also highlights the importance of considering regional variations in treatment protocols and microbial patterns, particularly in the context of the Turkish population studied. The differences in LOS associated with various treatments emphasize the need for localized data to inform FN management strategies. Effective management of febrile episodes and supportive therapies is crucial not only for reducing LOS but also for ensuring better resource allocation and patient care. These insights provide valuable guidance for improving FN outcomes and optimizing hospitalization practices. Furthermore, our results support the fact that tailored antibiotic selection and supportive care strategies are essential to improving clinical outcomes, minimizing healthcare resource utilization, and enhancing hospital efficiency.

During hospital treatment, LOS has been utilized as a proxy for patient well-being, and it is considered to reflect the severity of the disease and the patient’s health status influenced by the overall medical treatment, the standard of care, and long-term care facilities.^[15]^ Although the literature provides evidence linking FN to the length of hospital stay in cancer patients, significant variations in patient demographics, study designs, sample sizes, and assessment methods across studies make comparisons challenging. The relevant literature on the relationship between FN and LOS in cancer patients was reviewed with the aim of consolidating our findings and highlighting the most significant clinical implications while also identifying key directions for future research in this area. In most studies, a significant relationship was determined between LOS and neutropenia.^[10,12]^ Rosa et al investigated factors influencing hospital LOS in cancer patients with FN, identifying high-dose chemotherapy regimens, prolonged neutropenia, and bloodstream infections caused by Gram-negative multidrug-resistant bacteria as predictors of extended LOS in adult patients with hematologic neoplasms.^[10]^ In contrast, our study focused on patients with solid tumors. While these 2 studies examined FN in distinct patient populations, both highlight the significant role of prolonged febrile duration as a critical factor influencing hospital LOS, underscoring its importance in the management of FN. Daniels et al detected the impact of the timing of antibiotic use in the management of FN in their retrospective cohort study.^[12]^ They reported that timely antibiotic administration is crucial in the treatment of FN; however, moderate delays in initiating antibiotics did not significantly affect outcomes. In our clinical practice, oncology consultation services are provided promptly when patients present to our emergency unit, and antibiotic therapy is initiated without delay. Therefore, the impact of early or delayed antibiotic administration on hospital LOS was not evaluated in this study. Further investigation is required to determine whether delays in antibiotic administration serve as a predictor of LOS.

It is well known that an increase in hospital LOS is often attributed to patients who continue to exhibit fever and worsening signs and symptoms of infection, requiring prolonged hospitalization rather than being discharged.^[16]^ A 2019 Cochrane review reported that data on hospital LOS were insufficient to draw any useful conclusions.^[17]^ Furthermore, apart from its detrimental effect on overall survival and the quality of life, FN has pharmacoeconomic implications. Therefore, our study aimed to focus on achieving a precise understanding of the factors influencing hospital LOS, along with leveraging ongoing advancements in monitoring and process optimization to enable more effective management of inpatient LOS. We believe that the findings of our study will provide valuable insights for evaluating and improving treatment management during hospitalization processes.

In a study by Bachlitzanaki et al, the researchers examined the epidemiological and microbiological characteristics of hospitalized neutropenic patients with solid tumors, focusing on the duration of neutropenia and identifying factors influencing patient outcomes.^[3]^ They found that treatment duration was closely linked to risk assessment, with low-risk neutropenic patients requiring shorter treatment courses, often involving oral regimens, while high-risk patients required more intensive interventions. Additionally, their study emphasized that the length of hospital stay and patient outcomes were significantly affected by various risk factors, including the duration of neutropenia. Similarly, our findings align with this by highlighting the influence of risk factors such as febrile days, filgrastim administration, and antibiotic selection on hospital LOS. However, while Bachlitzanaki et al highlighted the role of neutropenia duration as a key factor, our study demonstrated the critical importance of managing fever duration and optimizing antibiotic regimens, particularly with the use of sulperazon, which significantly shortened LOS in our patient cohort. These differences may reflect variations in treatment protocols and study populations.

Chindaprasirt et al conducted an extensive study on adult cancer patients with FN, analyzing hospital LOS, mortality rates, and associated costs.^[18]^ In comparison to the findings in their study reporting a range of 6.5 to 9.2 days, our study observed a shorter mean LOS of 6.09 days. While the referenced study highlighted that younger patients under 40 years of age tended to require longer hospitalization, our results did not show a significant correlation between age and LOS. This discrepancy may be attributed to differences in patient demographics, treatment protocols, or healthcare settings. Additionally, current literature does not provide conclusive evidence that intensive treatment for infectious complications and lower mortality rates extend the length of hospital stay specifically in younger cancer patients with FN. While some studies have examined factors influencing LOS and mortality in cancer patients with FN, they do not specifically address the impact of age. In our study, factors such as febrile days and filgrastim administration were more prominent predictors of LOS, with antibiotic selection also playing a critical role, particularly the shorter LOS observed in patients treated with sulperazon; however, we did not detect a significant relationship between demographic characteristics such as age and gender. These findings may help in developing tailored management strategies specific to patient populations and treatment protocols.

The meta-analysis of 11 clinical studies demonstrated that cefoperazone-sulbactam has clinical efficacy comparable to other commonly used antibiotics, such as piperacillin-tazobactam and carbapenems, in the empirical treatment of FN.^[19]^ This aligns with our findings, where sulperazon (cefoperazone-sulbactam) was associated with a significantly shorter hospital LOS compared to meronem and tazocin. The meta-analysis also highlighted similar treatment success rates across pediatric and adult populations and a low pooled all-cause mortality rate of 6.0%, reinforcing the effectiveness of cefoperazone-sulbactam. In our study, the use of sulperazon as an empiric antibiotic not only reduced LOS but also demonstrated its potential for optimizing FN management without compromising clinical outcomes. These findings collectively emphasize the value of cefoperazone-sulbactam as a cost-effective and efficient alternative in FN treatment protocols.

To aid in the management of FN, numerous professional societies have established practice guidelines.^[1,20]^ These guidelines not only provide recommendations for empiric antibiotic therapy but also address more complex and expensive treatments, including the use of therapeutic granulocyte-colony stimulating factor, antifungal and antiviral agents, and empiric vancomycin. The study by Wright et al highlights the impact of filgrastim on reducing hospital LOS, with 33.8% of their cohort achieving a hospital stay of <3 days after receiving 1 to 2 days of filgrastim.^[21]^ In contrast, our findings reveal that prolonged filgrastim administration was associated with an increased LOS. Specifically, patients receiving filgrastim had a significantly longer median LOS compared to those who did not (5.00 vs 3.00 d, *P* = .042). This discrepancy may be attributed to differences in patient populations, the timing of filgrastim initiation, and the severity of FN episodes. While Wright et al’s study underscores the potential for early, short-duration filgrastim to optimize LOS, our results suggest that prolonged filgrastim use might reflect more severe or complicated cases requiring extended hospitalization. These findings highlight the importance of personalized treatment strategies and the need for further research to delineate the optimal use of filgrastim in FN management.

The etiological landscape of FN in adults is complex and varies by region, underlying malignancy, and prior antibiotic exposure. Gram-negative bacteria, particularly *Escherichia coli*, *Klebsiella pneumoniae*, and *Pseudomonas aeruginosa*, continue to be predominant pathogens in FN episodes, often associated with higher morbidity and mortality rates.^[1,10]^ In recent years, an alarming rise in multidrug-resistant organisms, including extended-spectrum β-lactamase-producing *Enterobacteriaceae* and carbapenem-resistant strains, has been documented in FN cohorts globally, necessitating critical reassessment of empirical antibiotic regimens.^[19,20]^ In Türkiye, studies have similarly reported a shift towards resistant Gram-negative pathogens, with ESBL-producing *E. coli* and *K. pneumoniae* being increasingly prevalent in oncology settings.^[6,7]^ These trends underscore the importance of tailoring empiric antimicrobial strategies to local resistance patterns. Our study’s findings, which demonstrated a significantly shorter LOS in patients treated with sulperazon (cefoperazone-sulbactam), may reflect the clinical efficacy of this agent against locally prevalent resistant pathogens. Therefore, evaluating antibiotic choices in FN management is essential not only for patient outcomes but also for combating emerging resistance threats in vulnerable cancer populations.

While our research primarily examined clinical factors such as the duration of fever, choice of antibiotics, and administration of filgrastim, it is crucial to recognize that the LOS is influenced by multiple factors. Non-clinical aspects, including discharge protocols, the presence of caregivers at home, the socioeconomic status of patients, and logistical challenges like bed availability or care coordination, may also impact LOS but were not included in our analysis. Furthermore, the type and stage of cancer could independently influence hospitalization requirements, regardless of the severity of infection. The absence of data regarding these potential confounding variables constitutes a limitation of our research. Future prospective studies that integrate both medical and contextual factors may provide a clearer understanding of the intricate relationships affecting LOS in cases of FN.

Our research has several limitations. Firstly, it is based on data gathered from a hospital-based, tertiary clinic. As one of the largest hospitals in the region, our tertiary care center serves as a referral hub for nonresponsive and advanced-stage patients. The intensive chemotherapy regimens administered to these patients may contribute to the observed long LOS. Additionally, the exclusion of certain patient groups, such as those with hematologic malignancies or identified sources of infection, may limit the generalizability of the results to broader FN populations. Despite these limitations, the strength of this study lies in its investigation of the relationship between hospital LOS and empiric antibiotic choice, setting it apart from other studies in the literature. To the best of our knowledge, this is the first study to specifically examine the impact of antibiotic selection on LOS in FN patients. To accurately assess this relationship, we sought to create a more homogeneous and specific patient group by excluding factors known to influence LOS, as highlighted in previous studies. Thus, patients with delayed antibiotic initiation (over 2 h), chronic organ failure, an identified source of infection, or positive blood culture results were excluded from our analysis. This approach allowed us to isolate and better evaluate the effect of empiric antibiotic choice on hospitalization outcomes.

## 5. Conclusion

This study highlights the relevance of considering regional variations in treatment protocols and patient outcomes in managing FN. Additionally, we demonstrated that empiric antibiotic choice, particularly the use of sulperazon, is significantly associated with shorter LOS, underscoring the critical role of tailored antibiotic regimens in FN management. To improve patient outcomes and resource utilization, these findings highlight the importance of early screening and intervention strategies targeted at optimizing treatment protocols and managing febrile episodes effectively. Future research should focus on elucidating the mechanisms linking antibiotic regimens and febrile management to adverse clinical outcomes, further advancing FN care practices.

## Acknowledgments

We would like to thank the participants and our department’s staff for their contributions to this study.

## Author contributions

**Conceptualization:** Ruhper Cekin, Sener Cihan.

**Data curation:** Ruhper Cekin.

**Formal analysis:** Ruhper Cekin.

**Investigation:** Ruhper Cekin, Didar Senocak.

**Methodology:** Ruhper Cekin.

**Resources:** Ruhper Cekin, Didar Senocak, Sener Cihan.

**Software:** Ruhper Cekin.

**Supervision:** Sener Cihan.

**Validation:** Ruhper Cekin.

**Writing – original draft:** Ruhper Cekin.

**Writing – review & editing:** Ruhper Cekin, Didar Senocak, Sener Cihan.
